# Human fibrosarcoma cells selected for ultra-high doxorubicin resistance, acquire trabectedin cross-resistance, remain sensitive to recombinant methioninase, and have increased c-MYC expression

**DOI:** 10.3389/fonc.2025.1704021

**Published:** 2025-12-08

**Authors:** Sei Morinaga, Qinghong Han, Kohei Mizuta, Byung Mo Kang, Michael Bouvet, Norio Yamamoto, Katsuhiro Hayashi, Hiroaki Kimura, Shinji Miwa, Kentaro Igarashi, Takashi Higuchi, Hiroyuki Tsuchiya, Satoru Demura, Robert M. Hoffman

**Affiliations:** 1 AntiCancer Inc., San Diego, CA, United States; 2 Department of Surgery, University of California, San Diego, San Diego, CA, United States; 3 Department of Orthopaedic Surgery, Graduate School of Medical Sciences, Kanazawa University, Kanazawa, Japan

**Keywords:** HT1080, ultra-high-doxorubicin-resistance, cross-resistance, sensitivity, trabectedin, recombinant methioninase, Hoffman effect, methionine restriction

## Abstract

**Background:**

Doxorubicin is standard first-line chemotherapy for soft-tissue sarcoma (STS), yet the emergence of resistance severely limits its clinical efficacy. Developing novel strategies to overcome resistance are critical for improving soft-tissue sarcoma patient outcomes.

**Methods:**

An ultra-high doxorubicin-resistant (UHDR) HT1080 fibrosarcoma cell line was established through stepwise exposure to increasing doxorubicin concentrations over 5-months. Over the course of the five months, HT1080 cells were cultured in doxorubicin concentrations that increased stepwise from 8 nM to 15 µM, an 1875-fold increase. Cell viability assays for HT1080 and UHDR were performed using the WST-8 cell-viability agent. c-MYC expression was analyzed by Western blotting.

**Results:**

UHDR-HT1080 cells exhibited an 11.6-fold increase in doxorubicin resistance compared with parental HT1080 cells and displayed selective cross-resistance to trabectedin (8.9-fold), while remaining sensitive to recombinant methioninase (rMETase). rMETase synergistically enhanced doxorubicin efficacy in UHDR cells. Western blotting demonstrated an 8.4-fold elevation in c-MYC expression in UHDR-HT1080 cells.

**Conclusion:**

The findings indicate that rMETase can overcome ultra-high doxorubicin resistance in fibrosarcoma cells, likely through targeting methionine addiction, a universal metabolic vulnerability of cancer. These results support the potential clinical application of methionine restriction therapy to treat doxorubicin-resistant STS.

## Introduction

Soft tissue sarcomas (STS) are a heterogeneous group of malignant tumors that arise from mesenchymal tissues and account for approximately 1% of all adult cancers (1). Doxorubicin is a first-line chemotherapy for STS (2). The development of acquired resistance to doxorubicin in STS progression has limited the clinical efficacy of this drug, resulting in a 5-year survival of only 8–9% 3, 4). High levels of c-MYC expression correlate with poor prognosis and increased resistance to chemotherapeutic agents in various cancer types, including STS (5, 6). Metastatic STS remains a recalcitrant disease in need of improved therapy especially to overcome resistance to first-line therapy.

Methionine addiction is a general and fundamental hallmark of cancer termed the Hoffman effect (7, 8). Methionine restriction, including recombinant methioninase (rMETase), selectively arrests cancer cells in the late-S/G_2_ phase of the cell cycle (9, 10) and has been shown to increase the efficacy of all types of chemotherapy drugs that target cells in late-S/G_2_ (11–18). Recently, we have studied the rMETase sensitivity of drug-resistant sarcoma cells (18–24).

Very few studies have focused on overcoming doxorubicin resistance of STS. The present study focused on exploiting the metabolic vulnerability of methionine addiction as a therapeutic target to overcome ultra-high doxorubicin-resistance in soft-tissue sarcoma by establishing an ultra-high doxorubicin-resistant cell model to test methionine-restriction-based strategies.

## Materials and methods

### Cell culture

The American Type Culture Collection (Manassas, VA, USA) provided the HT1080 cell line. Cells were grown in Dulbecco's modified Eagle's medium (DMEM) with 10% fetal bovine serum (FBS) and 1 IU/ml penicillin and streptomycin.

### Reagents

Bedford Laboratories (Bedford, OH, USA) provided doxorubicin. AntiCancer Inc. (San Diego, CA, USA) produced recombinant methioninase (rMETase). The process of producing rMETase has been published (8): Briefly, the *Pseudomonas putida methioninase* gene was cloned in *E. coli*, which was fermented to produce recombinant methioninase, which was purified with a heat step, polyethylene glycol precipitation, and diethylaminoethyl-sepharose fast-flow ion-exchange column chromatography (25).

### Establishment of an ultra-high doxorubicin-resistant HT1080 cells

Over the course of five months, HT1080 cells were cultured in doxorubicin concentrations that increased stepwise from 8 nM to 15 µM, an 1875-fold increase. The initial concentration of 8 nM approximated the lower cytotoxic threshold reported for HT1080 cells (18), while the final concentration of 15 µM was intentionally set above clinically-achievable plasma levels (approximately 3–6 µM) (26) to provide strong selective pressure for establishing ultra-high resistant subclones. Because the present study aimed to develop an *in vitro* ultra-high resistance model rather than reproduce clinical pharmacokinetics, the chosen range was determined based on *in vitro* cytotoxicity data rather than plasma-exposure levels.

### IC_50_ determination for doxorubicin and rMETase

Cell viability was assessed using the WST-8 reagent (Dojindo Laboratory, Kumamoto, Japan). HT1080 or UHDR-HT1080 cells were cultured in 96-well plates at a concentration of 3×10^3^ cells per well in DMEM (100 μl/well). After that, the plates were incubated overnight at 37°C. The cells were treated for 72 hours with either rMETase at concentrations ranging from 0.5 U/ml to 8 U/ml or doxorubicin at concentrations ranging from 1 µM to 40 µM. Each well received 10 μl of the WST-8 solution following the culture period. The plates were then incubated at 37°C for an additional hour. In a microplate reader (SUNRISE: TECAN, Mannedorf, Switzerland), the absorption of cells treated with WST-8 was measured at 450 nM. Microsoft Excel for Mac 2016 version 15.52 (Microsoft, Redmond, Washington, United States) was used to create the drug sensitivity curves. ImageJ version 1.53k (National Institutes of Health, Bethesda, MD, USA) was used to calculate the half-maximal inhibitory concentration (IC_50_) values. IC_50_ values were derived from nonlinear regression curves fitted using ImageJ-generated absorbance data normalized to untreated controls. All assays were performed within 72 h after drug preparation to minimize degradation and ensure consistent potency. Each experiment was carried out twice, in triplicate.

### Doxorubicin–rMETase combination drug-sensitivity assay

96-well plates were seeded with 3×10^3^ UHDR-HT1080 cells. The cells received the following treatment after 24 hours: 1) No treatment; 2) Doxorubicin alone; 3) rMETase alone; and 4) the combination of rMETase and doxorubicin. After 72 hours, cell viability was determined using the WST-8 reagent in triplicate as described above. Bliss analysis was also performed to determine whether the combined treatment produced synergistic, additive, or antagonistic effects. Cell viability was normalized to the untreated control (set at 100%), and inhibition rates were calculated as 1 – (viability/100). The expected additive inhibition (E_Bliss_) was computed using the formula E_Bliss_ = E_A_ + E_B_ – (E_A_× E_B_), where E_A_ and E_B_ represent the fractional inhibitions of doxorubicin and rMETase, respectively. The difference between the observed combined inhibition (E_AB_) and E_Bliss_ was defined as ΔBliss = E_AB_ – E_Bliss_. A positive ΔBliss indicated synergy; a value near 0, additivity; and a negative ΔBliss, antagonism (27).

### Cross-resistance assay

The same protocol as in drug sensitivity assay 1 was used. Second-line drugs for soft tissue sarcoma, eribulin (Eisai Inc., Nutley, NJ, USA) (0.5–8 nM), trabectedin (PharmaMar, Horsham, PA, USA) (1–40 nM), gemcitabine (BluePoint Laboratories, Little Island, Cork, Munster, Ireland) (4–64 nM), and docetaxel (Accord Healthcare Inc., Durham, NC, USA) (1–16 nM), were used to determine cross-resistance of UHDR-HT1080 cells. The IC_50_ values of each drug for HT1080 and UHDR-HT1080 were determined. Cross-resistance to a drug was indicated when there was an increase of 2-fold or more in the IC_50_.

### Western immunoblotting

Proteins were extracted from HT1080 and UHDR-HT1080 cells using RIPA Lysis Buffer and Extraction Buffer (Thermo Fisher Scientific, Waltham, MA, USA) and 1% Halt Protease Inhibitor Cocktail (Thermo Fisher Scientific). 10% SDS-PAGE gels were loaded with protein samples. The samples were then transferred to polyvinylidene difluoride (PVDF) membranes with a thickness of 0.45 μm (GE Healthcare, Chicago, IL, USA). Membrane blocking was done using Bullet Blocking One for Western Blotting (Nakalai Tesque, Inc., Kyoto, Japan). The anti-c-MYC antibody was obtained from Proteintech (1:2,000, #10828-1-AP, Rosemont, IL, USA) as well as β-Actin (20536-1-AP, 1:1,000). Horseradish-peroxidase–conjugated anti-rabbit IgG (1:5,000, #SA00001-2, Proteintech) antibody was used as the secondary antibody. The western blot was scanned using the Clarity Western ECL Substrate (Bio-Rad Laboratories, Hercules, California, USA) and UVP ChemStudio imaging machine (Analytik Jena, Upland, CA, USA). Band intensities were quantified using ImageJ software (version 1.53k, NIH). The relative c-MYC expression level was normalized to β-actin, and the ratios were calculated from three independent experiments.

Statistical analyses were conducted using EZR software (Jichi Medical University, Saitama, Japan) (28). The Welch’s t-test and Tukey-Kramer analysis were employed to ascertain the correlation between the variables. Statistically significant *p*-values were defined as less than 0.05 (29).

## Results

### Establishment of ultra-high doxorubicin-resistant HT1080 cells

**Figure 1:**
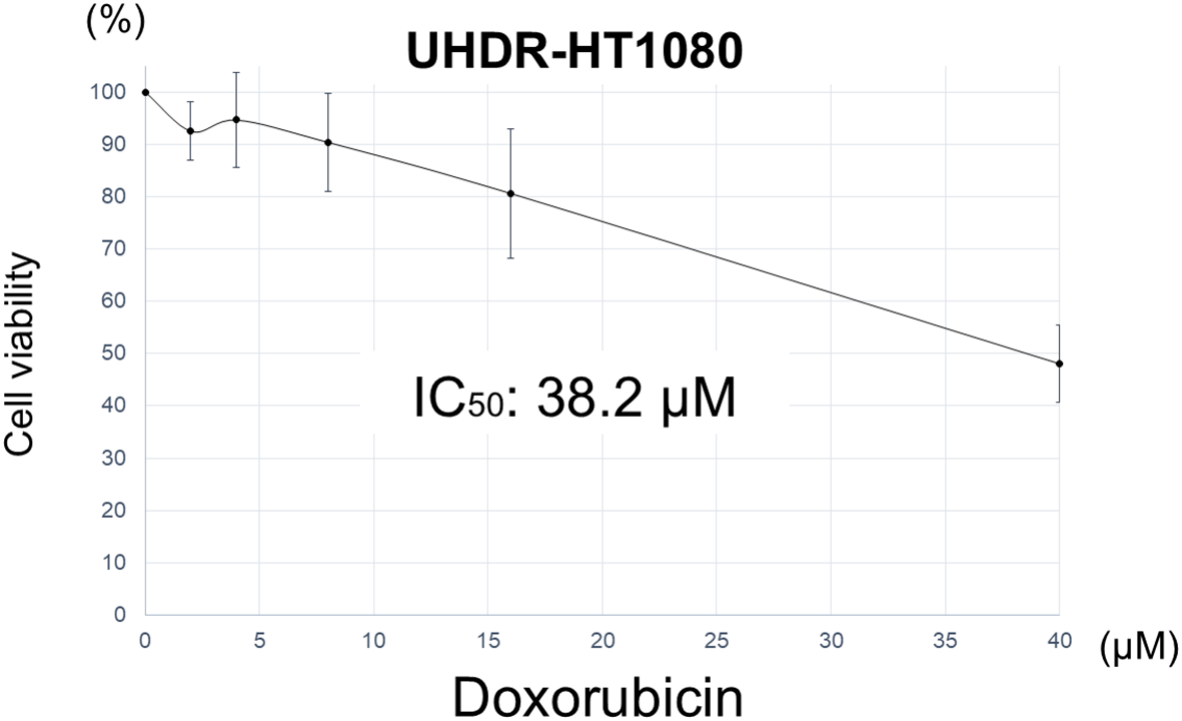
IC_50_ of doxorubicin on UHDR-HT1080 cells. Please see Materials and Methods for details. Results are shown as mean ± standard deviation.

Ultra-high doxorubicin-resistant cells (UHDR-HT1080) were selected from HT1080 cells by culturing them in doxorubicin, increasing the concentration stepwise from 8 nM to 15 µM, an 1875-fold increase over a period of 5 months. The HT1080 IC_50_ of doxorubicin was 3.3 µM [data from (18)], compared to 38.2 µM for UHDR-HT1080 cells that were finally selected. UHDR-HT1080 cells were 11.6 times more resistant to doxorubicin than the parental HT1080 cells (**Figure 1**).

### Determination of IC_50_ of rMETase alone on HT1080 and UHDR-HT1080

**Figure 2:**
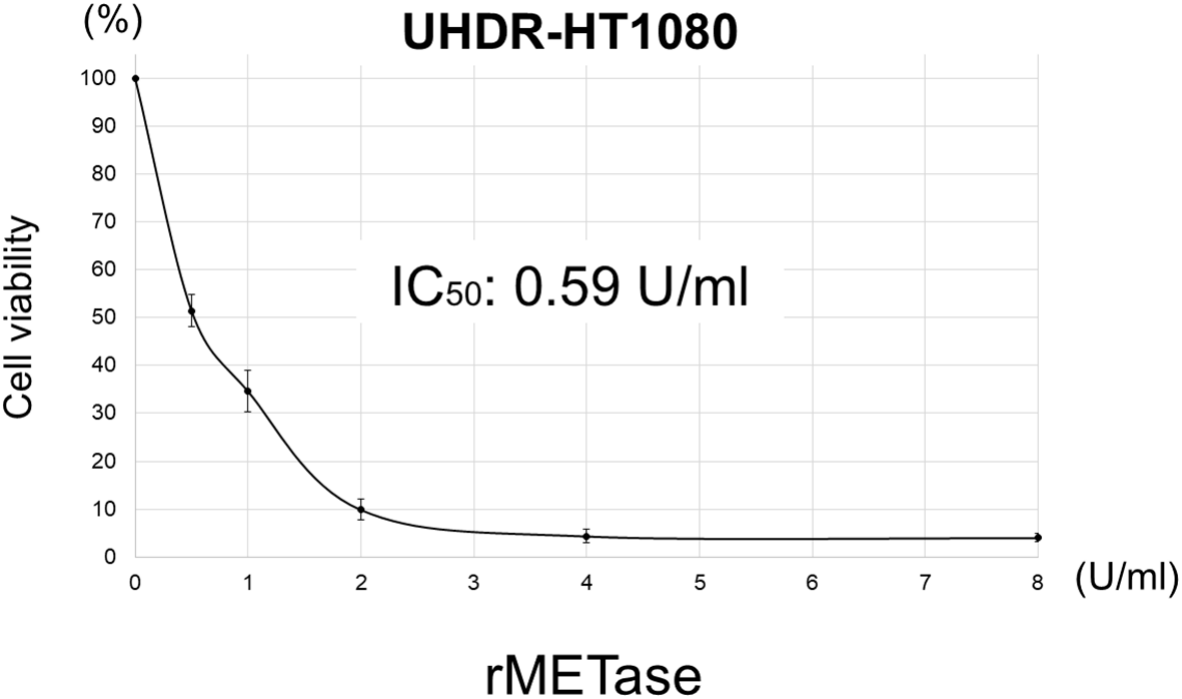
IC_50_ of rMETase on UHDR-HT1080 cells. Please see Materials and Methods for details. Results are shown as mean ± standard deviation.

The HT1080 IC_50_ of rMETase was 0.75 U/ml [data from (12)], compared to the UHDR-HT1080 IC_50_ of 0.59 U/ml (**Figure 2**).

### Cross-resistance of UHDR-HT1080 cells to second-line STS chemotherapy drugs

The IC_50_ values ​​for HT1080 and UHDR-HT1080 cells were 0.15 nM [data from (12)]and 0.28 nM for eribulin, respectively; 3.3 nM [data from (17)] and 29.3 nM for trabectedin, respectively; 12.8 nM [data from (29)] and 13.6 nM for gemcitabine, respectively; and 1.68 nM [data from (21)] and 1.83 nM for docetaxel, respectively (**Table 1**). Of these drugs tested, only trabectedin showed cross-resistance in UHDR-HT1080cells with an 8.9-fold increase in the IC_50_.

**Table 1:** IC_50_ of HT1080 and ultra-highly doxorubicin-resistant HT1080 (UHDR-HT1080) cells for eribulin, trabectedin, gemcitabine or docetaxel.

	HT1080 (IC_50_)	UHDR-HT1080 (IC_50_)
Eribulin (nM)	0.15 [data from (12)]	0.28
Trabectedin (nM)	3.3 [data from (17)]	29.3
Gemcitabine (nM)	12.8 [data from (29)]	13.6
Docetaxel (nM)	1.68 [data from (21)]	1.83

UHDR-HT1080 cells had cross-resistance to trabectedin. Cross-resistance is defined as >2-fold increase in IC_50_.

### Efficacy of rMETase combined with doxorubicin on UHDR-HT1080 cells

**Figure 3:**
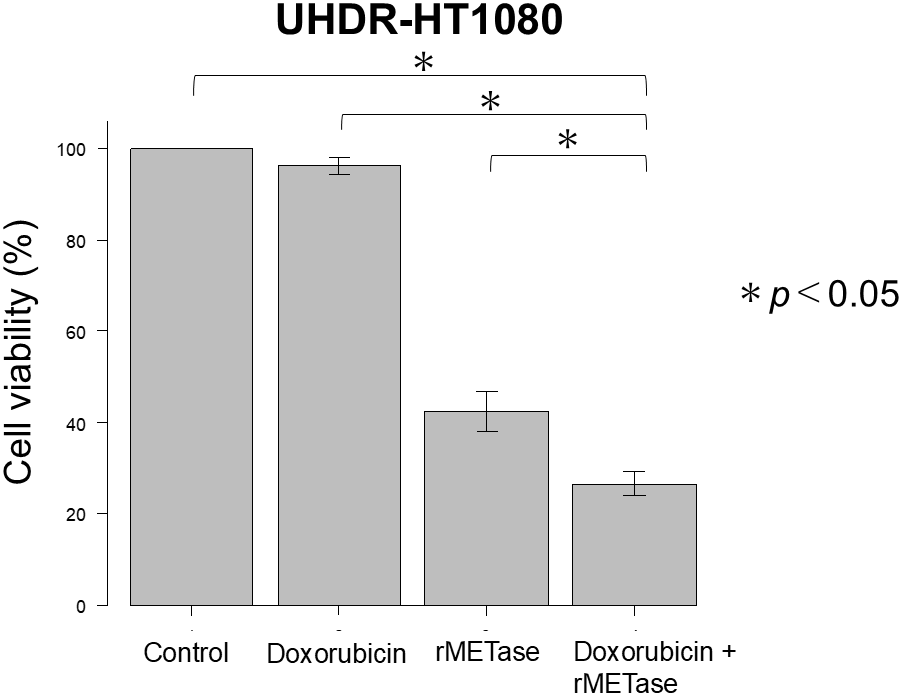
rMETase sensitized ultra-high doxorubicin-resistant HT1080 (UHDR-HT1080) fibrosarcoma cells to doxorubicin. Control (DMEM); doxorubicin [3.3 μM (IC_50_ of HT1080)]; rMETase [0.59 U/ml (IC_50_ of UHDR-HT1080)]; doxorubicin [3.3 μM (IC_50_ of HT1080)] plus rMETase [0.59 U/ml (IC_50_ of UHDR-HT1080)]. doxorubicin [3.3 µM (IC_50_of HT1080)] and rMETase [0.59 U/ml (IC_50_ of UHDR-HT1080)]. Data are shown as mean ± standard deviation. Please see Materials and Methods for details. The asterisk (*) indicates statistical significance at p < 0.05.

The IC_50_ of rMETase for UHDR-HT1080 (0.59 U/ml) combined with the IC_50_ of doxorubicin for HT1080 (3.3 µM) inhibited UHDR cells 73.4% compared to the untreated control; 69.7% compared to doxorubicin alone; and 15.8% compared to rMETase alone (*p* < 0.05) (**Figure 3**). The expected additive inhibition calculated from the Bliss model was 59.2%. The observed inhibition exceeded this value by ΔBliss = +14.2%, indicating a synergistic interaction between rMETase and doxorubicin.

### Western blotting of c-MYC

**Figure 4:**
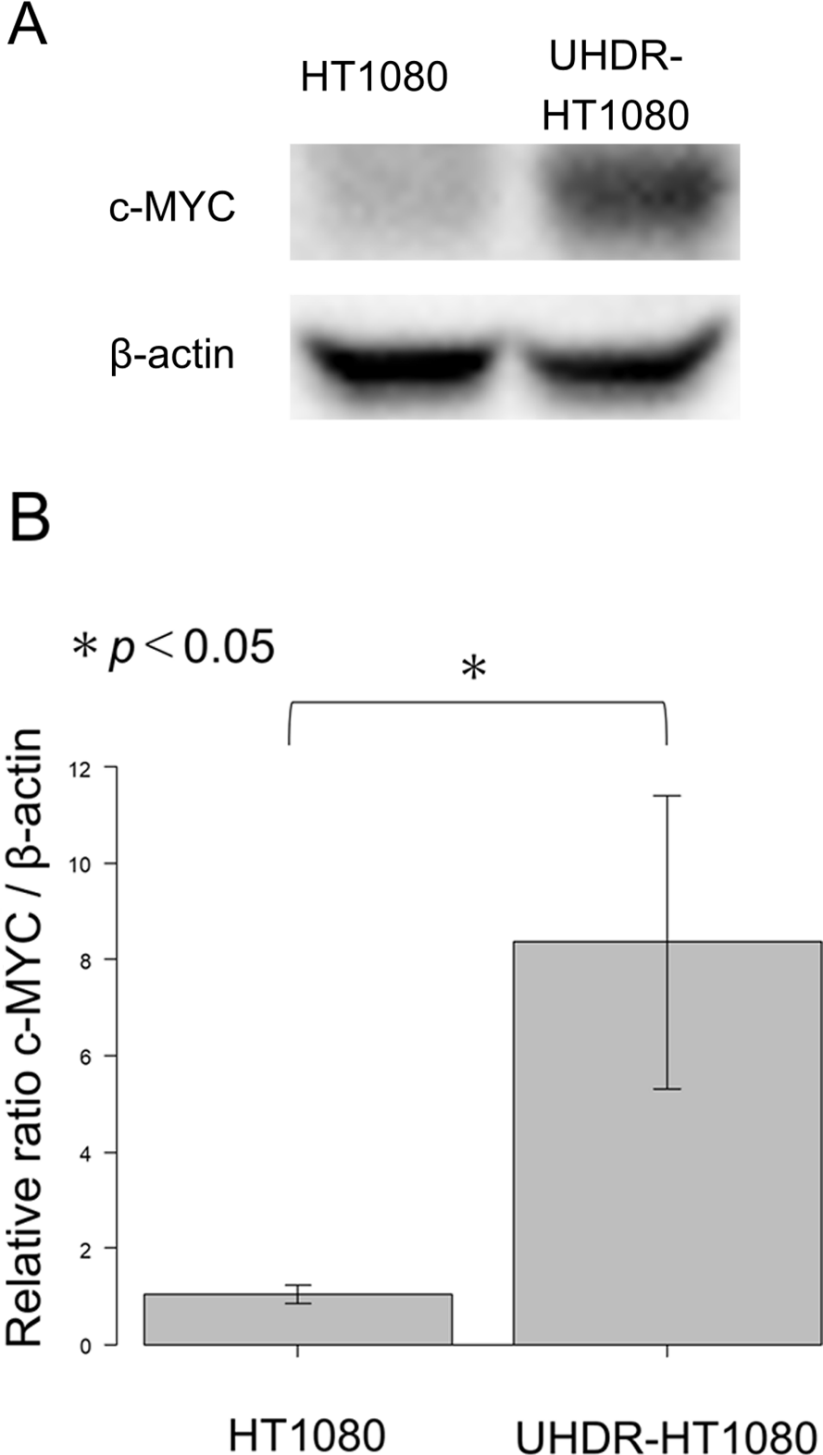
Expression of c-MYC in HT1080 and ultra-high doxorubicin-resistant HT1080 (UHDR-HT1080) fibrosarcoma cells. **(A)** Western blot of c-MYC expression in HT1080 and UHDR-HT1080 cells. Quantitative densitometry results from three independent experiments are now included in **(B)**. Bar graphs show an 8.4-fold increase in c-MYC expression normalized to β-actin in UHDR-HT1080 compared to HT1080 cells. Quantitative densitometry results from three independent experiments are now included in **(B)**, showing an 8.4-fold increase in c-MYC expression normalized to β-actin. Data shown are representative of three different Western blots. Please see materials and methods for details. The asterisk (*) indicates statistical significance at p < 0.05.

c-MYC expression in UHDR-HT1080 cells increased 8.4-fold compared to HT1080 cells (*p* < 0.05) (**Figure 4**).

## Discussion

UHDR-HT1080 was established by selecting HT1080 cells in stepwise increasing concentrations of doxorubicin (1875-fold) over five months. UHDR-HT1080 cells acquired ultra-high resistance to doxorubicin, with an IC_50_ value of 38.2 µM, which is 11.6-fold greater than that of parental HT1080 cells. Future experiments will examine whether the resistance persists in doxorubicin-free culture.

UHDR-HT1080 cells were cross-resistant to second-line therapy, trabectedin by 8.9-fold, suggesting a shared resistance mechanism. The cross-resistance seen in UHDR-HT1080 cells to trabectedin may be due to many factors, resulting from a combination of enhanced DNA repair, drug efflux, and modified cell cycle regulation (30, 31), which will be studied in the future.

The IC_50_ of rMETase was similar in HT1080 and UHDR-HT1080, which indicates the acquisition of ultra-high doxorubicin resistance over many steps and five months did not affect rMETase sensitivity. rMETase sensitized UHDR-HT1080 cells 19.8-fold to doxorubicin. These results suggest that rMETase may be effective clinically to overcome doxorubicin resistance in STS. rMETase has been previously shown to be selectively cytotoxic to cancer cells but not normal fibroblasts (12, 13, 17, 18, 21, 32–41). rMETase is thermo-stable at least to 60°C (25).

We previously established moderately doxorubicin-resistant HT1080 cells (IC_50_ = 12.4 µM) which maintained sensitivity to rMETase (18). The present study characterized ultra-high doxorubicin-resistant HT1080 cells (IC_50_ = 38.2 µM) which still maintained rMETase sensitivity, which suggests the potential of rMETase to overcome clinical doxorubicin resistance in STS.

The sustained efficacy of rMETase in UHDR-HT1080 cells may be attributed to methionine addiction, a fundamental metabolic abnormality of cancer known as the Hoffman effect (7, 8). Methionine depletion causes arrest of cancer cells in the late-S/G_2_ phase, possibly enhancing the cytotoxicity of chemotherapeutic agents targeting DNA replication, such as doxorubicin (9, 10). Previous studies have shown that rMETase enhances the sensitivity of drug-resistant HT1080 human fibrosarcoma and 143B osteosarcoma cells to eribulin, trabectedin, gemcitabine, and docetaxel *in vitro* (19–22, 29). rMETase significantly enhanced the cytotoxic effects of each agent in drug-resistant soft-tissue sarcoma models (19–22, 29). In eribulin-resistant HT1080 cells, the combination of rMETase and eribulin achieved a synergistic reduction in cell viability, indicating methionine depletion reverses eribulin resistance (19). Similarly, rMETase was shown to increase the anti-cancer efficacy of trabectedin in both parental and resistant fibrosarcoma cells by targeting methionine addiction, thereby overcoming acquired resistance (20). In another study, rMETase combined with docetaxel significantly enhanced cytotoxicity in docetaxel-resistant HT1080 cells (DTR-HT1080), achieving a 7-fold increase in efficacy compared to docetaxel alone, while sparing normal fibroblasts (21). These results further establish that rMETase specifically sensitizes chemotherapy-resistant cancer cells without increasing toxicity to normal cells. In osteosarcoma models, rMETase synergistically reversed high-docetaxel resistance developed in 143B cells (22). Gemcitabine-resistant HT1080 cells had elevated c-MYC expression but maintained high sensitivity to rMETase. rMETase restored gemcitabine responsiveness in resistant cells, possibly through cell cycle arrest in S/G_2_ phase of the cancer cells, where gemcitabine exerts its effect (29). rMETase has been shown to be effective in cancer patient-derived orthotopic xenograft (PDOX) mouse models (42). In the present study, rMETase synergistically increased the sensitivity of ultra-high doxorubicin-resistant HT1080 cells to doxorubicin. These findings indicate that rMETase overcomes chemotherapy resistance among a broad spectrum of agents by targeting the universal hallmark of methionine addiction in cancer cells (8). rMETase may thus serve as a promising adjunctive strategy in treating recalcitrant sarcomas that have failed standard therapies.

c-MYC is a transcription factor involved in cancer cell proliferation, and its expression has been associated with chemoresistance (5, 43). In osteosarcoma, elevated c-MYC expression has been linked to resistance to both doxorubicin and methotrexate (44, 45). However, no studies to date have investigated the relationship between c-MYC expression and doxorubicin resistance in soft tissue sarcomas. In the present study, we observed increased c-MYC expression in ultra-high doxorubicin-resistant HT1080 cells.

Further studies will also determine if c-MYC overexpression is linked to ultra-high doxorubicin resistance in STS, as well as the mechanism of cross-resistance to second-line STS chemotherapy in UHDR cells.

The present results suggest that combining rMETase and doxorubicin may be a clinical strategy to overcome clinical doxorubicin-resistant STS that are also cross-resistant to second-line STS drugs.

## Data Availability

The raw data supporting the conclusions of this article will be made available by the authors upon reasonable request.
